# Supplementary material for: Genetic Basis of Gap Formation Between Migrating Helicobacter pylori Colonies in Soft Agar Assays
Source: Microorganisms. 2025 May 7;13(5):1087. doi: 10.3390/microorganisms13051087 (PMC12114501; doi:10.3390/microorganisms13051087)
Supplement: Supplementary file 1 [file microorganisms-13-01087-s001.zip › microorganisms-3593809-supplementary.pdf]

Table S1. Primers used to locate Tn-7 insertion in merging mutants from Salama et al., 2004.

Primer Name	Sequence
CAT Tn7 N	ACTTTATTGTCATAGTTTAGATCTATTTT
CAT Tn7 S	ATAATCCTTAAAAACTCCATTTCCACCCC
CAT Tn7 N2	TCAGTTTAAGACTTTATTGTC
CAT Tn7 S2	CAGTTCCCAACTATTTTGTCC
Random A	GGCCACGCGTCGACTAGTACNNNNNNNNNNAGAG
Random B	GGCCACGCGTCGACTAGTACNNNNNNNNNNACGCC
Random C	GGCCACGCGTCGACTAGTACNNNNNNNNNNGATAT
Random PCR 2	GGCCACGCGTCGACTAGTAC

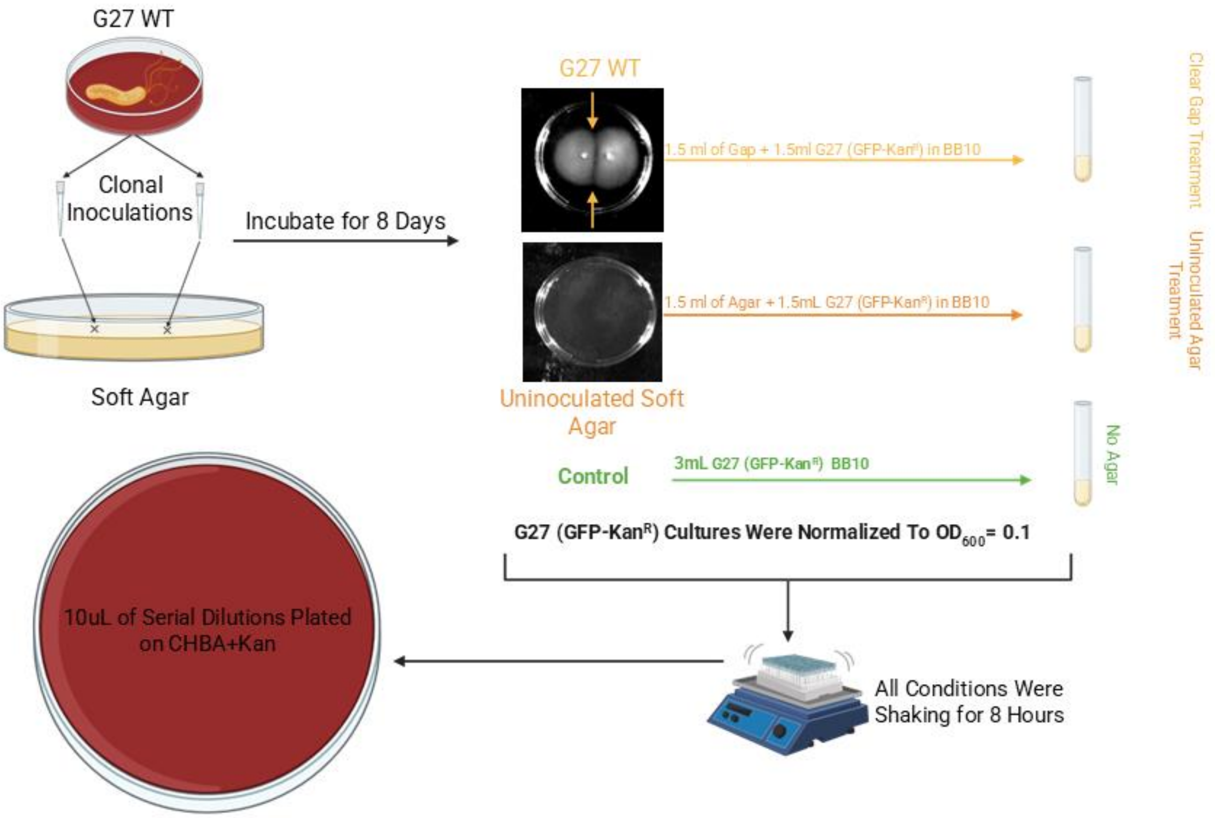

Supplemental Figure S1. Clear gap treatment growth curve experiment schematic. Clear gaps were extracted from G27 clonal WT colonies in competitive motility assays incubated for 8 days to ensure maximum concentrated extracts. Control conditions for comparison to the gap treatment include uninoculated agar incubated for 8 days and cultures grown

without any agar just liquid media. A total of 1.5mL of agar was accumulated and G27 GFP+ (KanR) culture and BB10 media were added to create 3mL cultures that were incubated for 8 hours. G27 GFP+ (Kan^R) inoculums were normalized OD600 of 0.1 initially. The final cultures were serially diluted and spot plated to screen for differential growth.

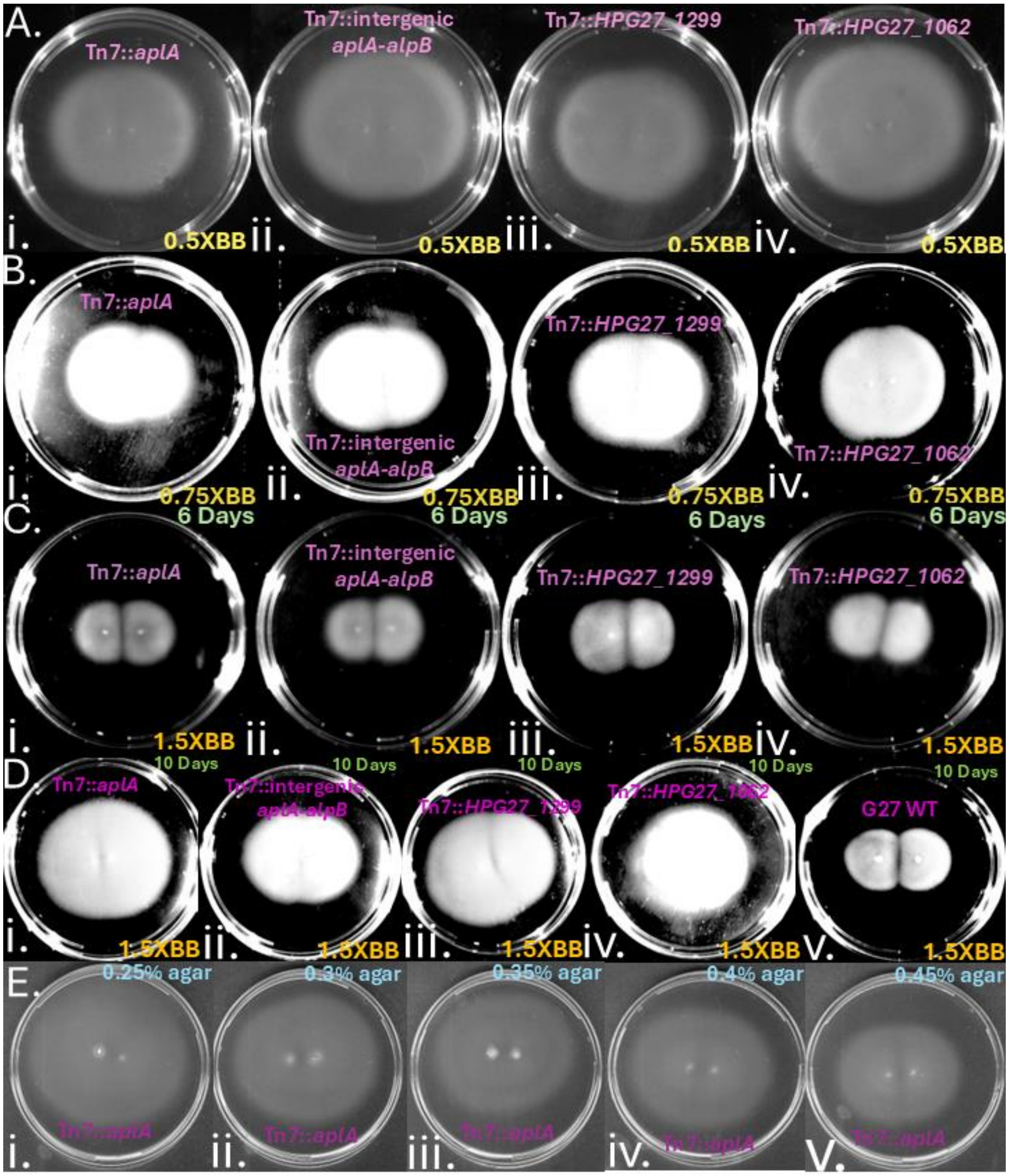

Supplemental Figure S2. Merge mutant phenotypes are not affected by Soft Agar Nutrient and Agar Components. Gap formation in transposon mutants is independent of nutrient availability and agar percentage. **(A)** Gap formation in soft agar with the indicated transposon mutants or control in (A)0.5X Brucella broth after 7 days, (B) 0.75X Brucella broth after 7 days or 1.5X Brucella broth after (C) 6 days or (D) 10 days. (E) Variation of agar concentrations in soft agar assays. Shown is the G27 Tn7::*alpA* but all other strains also displayed similar agar concentration-independent merge phenotypes.

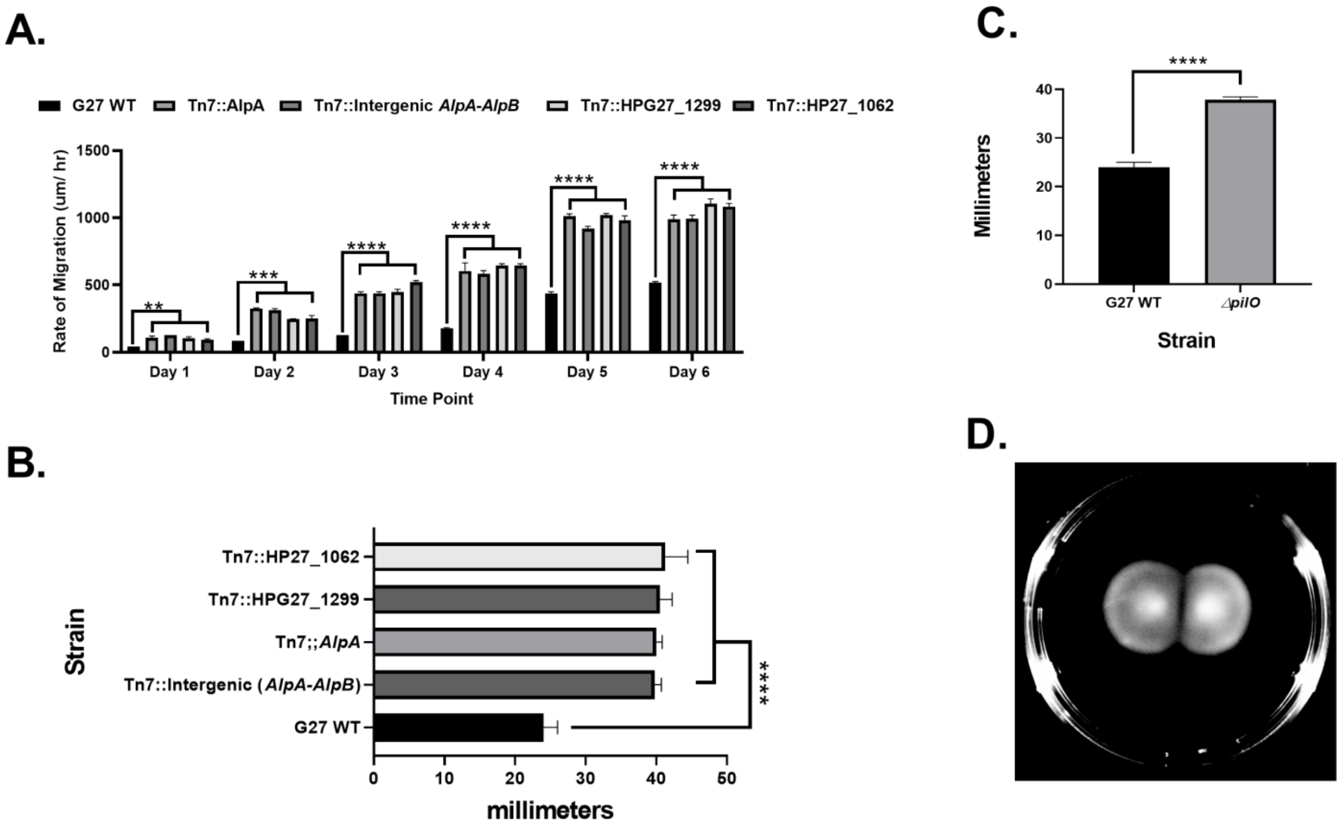

Supplemental Figure S3. Faster migration rates are not sufficient for gap formation. Soft agar migration rate was determined by measuring colony diameter over time. **(A)** G27 transposon isolates and wild type strains were grown non-competitively as single inoculations for six days in standard motility conditions. **(B)** Final diameter of migration for G27 transposon isolates were larger than the WT at 6 Days. **(C)** Final diameter of migration of the fast expanding G27 *ΔpilO::cat* transposon mutant **[13]** at 6 Days in non-competitive inoculations, **(D)** G27 *ΔpilO::cat* mutant inoculations form gaps.

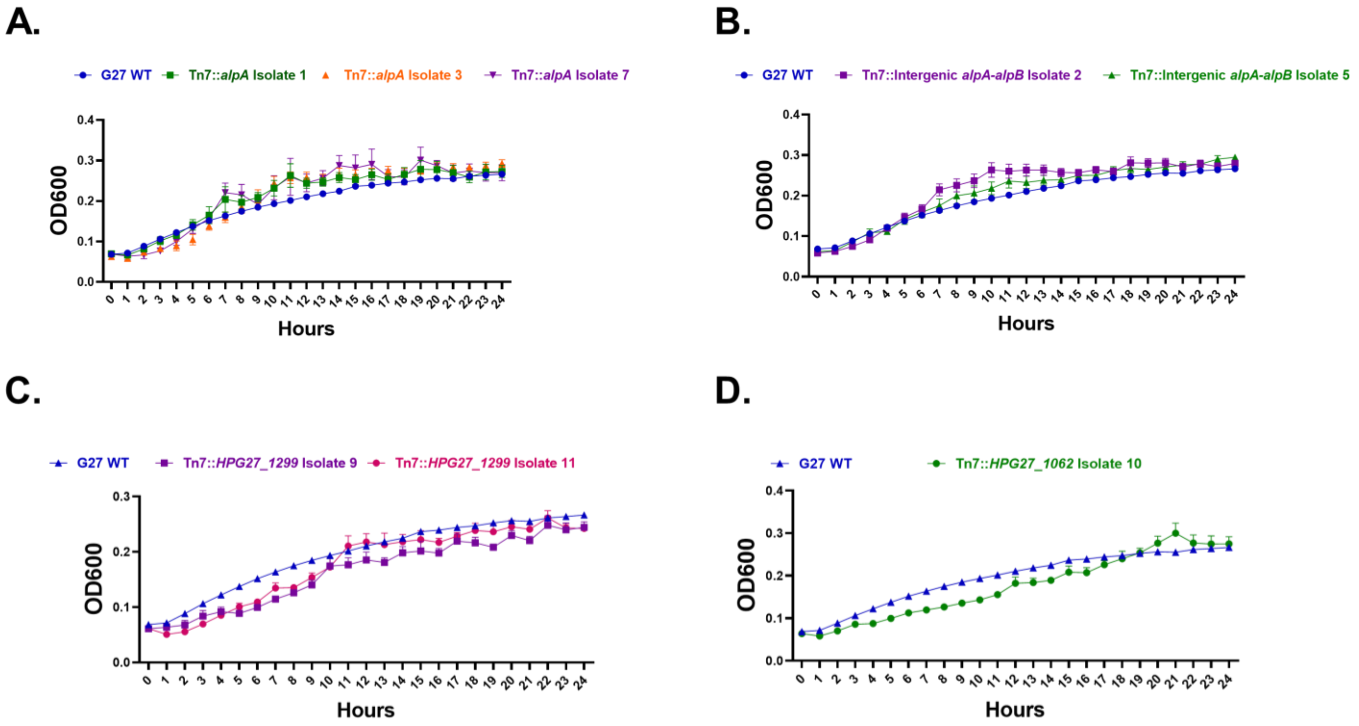

Supplemental Figure S4. Merge Tn-7 Mutants Planktonic growth in Liquid BB media with 2.5% FBS is similar to G27 WT. Planktonic growth curves with 2.5% FBS were conducted with Tn-7 mutants with merging phenotype to determine if differential cell division rate contribute to the absence of gaps of clearance.(i) Tn7::alpA, (ii) Tn7::Intergenic alpA- alpB, (iii) Tn7::HPG27_1299 (iv.) Tn7::HPG27_1062. Mutant grow similarly in 2.5% FBS as G27 WT.
